# Creep Compliance of Carbon Black-Filled Rubber Converted from Storage Modulus by Use of Collocation Method: Numerical and Experimental Validation

**DOI:** 10.3390/polym17131809

**Published:** 2025-06-28

**Authors:** Bo Zhou, Bin Zhao, Wei Tang, Rongyong Wang, Boyuan Yin

**Affiliations:** 1 Zhuzhou Times New Material Technology Co., Ltd., Zhuzhou 412000, China; zhoubo2004@csrzic.com (B.Z.); tangwei6@csrzic.com (W.T.); wangrongyong@csrzic.com (R.W.); 2 School of Civil Engineering, Hunan University of Science and Technology, Xiangtan 411201, China

**Keywords:** CB-filled rubber, generalized Kelvin model, collocation method, storage modulus, creep compliance

## Abstract

Carbon black (CB)-filled rubber has been widely used in engineering. However, its time-dependent behavior, such as creep, is undesirable during the service process. In addition, the long-term creep test is time- and cost-consuming. To this end, the objective of this paper aims to predict the creep behavior from the short-term storage modulus by use of the collocation method. First, the master curve of storage modulus was constructed based on the time–temperature superposition principle (TTSP), and the validation of shift factors was verified by use of the Williams–Landel–Ferry (WLF) equation. Second, the generalized Kelvin model was used to describe the master curve of storage modulus by use of the collocation method, and the corresponding parameters were obtained. Compared with the existing works, the collocation method had the advantages of avoiding the occurrence of waviness of the fitting curve. Lastly, the creep compliance of CB-filled rubber was calculated by substituting the fitting parameters into the creep compliance expression. In order to verify the reliability of the calculation result, the creep tests were carried out. It was obvious that the calculation result is in good agreement with the experimental one with a RMSE value of 0.0055, which means that the calculation result is reliable.

## 1. Introduction

Rubber material is a kind of extensively investigated material because of its excellent mechanical properties, which has been widely used in many engineering fields [1]. In the tire industry, styrene butadiene rubber (SBR) is an important elastomer, which has been widely used to enhance the compound characteristics of tires [2]. In the field of construction, Acrylonitrile butadiene rubber (NBR) is often used for the preparation of eco-friendly composites [3]. In toy production, natural rubber (NR) has become an important component [4]. In addition, it has been reported that rubber-based thermoplastic composites can offer high chemical resistance, greater flexibility and high impact strength [5]. In the actual service process, creep behavior is worthy of study for engineering because the rubber component often sustains loads for a long period [6]. When a constant load is applied to the rubber component, the deformation increases with the increase of loading time, which is known as creep behavior [7,8]. However, in the process of the creep test, the applied stress is difficult to keep constant because the cross-sectional area of the tested specimen decreases with the increase of loading time, especially for the long-term creep test. In addition, the long-term creep test is cost- and time-consuming. Therefore, an effective and reliable method to predict the creep behavior based on the short-term mechanical property is meaningful to scholars and engineers.

In order to investigate the creep behavior of rubber material, many works have been achieved from the aspects of the mechanism and constitutive model. As for the creep mechanism, many scholars pointed out that the creep behavior ascribed to the viscoelastic mechanism resulted from molecular disentanglement [9] and molecular slipping [10]. Similarly, Cui [11] investigated the effects of filler content and temperature on the creep behavior of rubber material and found that the creep process was attributed to the physical processes, such as molecular untangling. In addition, Banerjee [12] investigated the relationship between the creep behavior and the crosslink density of rubber material. The results showed that the creep resistance was greater when the rubber material had higher crosslink density, which had been confirmed by the reference [13]. As for the constitutive model, dashpots and springs are commonly combined in various ways, such as the Kelvin model and standard linear solid model [14]. Zhang [15] employed the Kelvin model to describe the creep behavior of reinforced rubber sealing composite and found that the calculation results are in accordance with the experimental ones. Traditionally, in order to broaden the application range of the above models, the generalized model, such as generalized Kelvin model, is a great choice [14]. In addition, the fractional model has been widely used to describe the viscoelastic properties of rubber material. Cai [16] developed a fractional rheological model to characterize the creep behavior, and the relationship between material parameters and temperatures was established. Meanwhile, in the process of integrating the constitutive equation, the characterization of the viscoelastic properties of rubber material using the fractional model is rather complicated [17].

As for the long-term creep behavior, the direct experimental investigation is cost- and time-consuming. In the long-term measuring of static tests, it has been reported that there are apparent deficiencies, such as the compliance of testing equipment, which can result in unreliable test data and affect the accuracy of the fitted Prony series parameters [18]. Generally, the time–temperature superposition principle (TTSP) is a useful method to determine the creep behavior in a wide range of temperature and time by shifting the experimental curves to construct a master curve. For example, Mandlekar [19] investigated the long-term creep behavior under various temperatures, and the Williams–Landel–Ferry (WLF) equation was modelled to verify the reliability of the TTSP. In addition, because of the higher experimental accuracy of the dynamic mechanical analysis (DMA) test in the laboratory, the static viscoelastic properties can be predicted by dynamic modulus on the basis of conversion methods [20].

In the conversion process, the collocation method is very useful for the prediction of the long-term static viscoelastic properties [21]. However, most existing works investigating the conversion of static viscoelastic properties from dynamic modulus mainly focus on other viscoelastic materials, such as asphalt mixture [18,22]. As for rubber material, the conversion of creep behavior from a dynamic modulus is open to question, which may be enhanced by adapting techniques used for other viscoelastic materials, such as asphalt or polymers.

Before the conversion process, the short-term DMA test should be carried out, and the dynamic master curve should be constructed by the use of the TTSP. Then, the dynamic master curve will be modelled by an appropriate viscoelastic model in order to obtain the material parameters. Nowadays, the generalized Kelvin model and generalized Maxwell model have been confirmed to be the effective models in the description of the dynamic master curve [23]. However, there are two disadvantages [24]: The fitting curve’s waviness, which does not satisfy the nature of the dynamic/relaxation modulus; Negative fitting parameters, which are against the physical significances. In the previous work [25], the relaxation modulus has been converted from the storage modulus, which verifies the reliability of the collocation method in avoiding the above two disadvantages and provides feasibility for this paper. Therefore, the conversion of creep behavior from dynamic modulus based on the collocation method is worthy of study, which is very important to the prediction of the long-term creep behavior of CB-filled rubber.

To this end, this paper aims to establish a quick and reliable conversion method of creep behavior of CB-filled rubber based on the dynamic master curve. Firstly, short-term DMA tests under various temperatures and frequencies were carried out, and the dynamic master curve at a reference temperature was constructed by the use of the TTSP. Secondly, the dynamic master curve was modelled by the generalized Kelvin model based on the collocation method, and the material parameters were obtained. Finally, the converted creep compliance was calculated by the creep compliance equation derived from the generalized Kelvin model, and the calculated creep compliance was verified by the creep testing results. The novelty of this paper is to investigate the interconversion between creep compliance and storage modulus by the use of the collocation method in order to avoid the above-mentioned disadvantages.

## 2. Materials and Methods

### 2.1. Materials

The materials used for DMA tests and creep tests are CB-filled rubber, which are provided by Zhuzhou Times New Material Technology Co., Ltd. Zhuzhou, China. The formulation is as follows: 100 phr NR (Thailand RRS3), 30 phr carbon black (N774), 6 phr antioxidant, 5 phr zinc oxide, 2 phr sulfur, 2 phr stearic acid, 2 phr micro crystal wax and 2 phr solid coumarone resin. The tested specimens have the dimensions of 35 × 5 × 2 mm^3^. Before the DMA tests and creep tests, all specimens were pre-stretched to exclude the Mullins effect.

### 2.2. DMA Tests

The DMA tests, based on the standard ISO 4664-1:2022 [26], were carried out by the use of a Gabo Eplexor 500N machine in a tensile mode. The prestrain was 1% and the dynamic strain amplitude was 0.2%. It has been reported that the testing strain range could keep the DMA tests in the range of linear viscoelasticity [14]. The testing frequency ranged from 0.1 to 80 Hz under different temperatures, i.e., −60, −50, −40, −30, −20, −10, 0, 10, 20, 30, 40, 50 and 60 °C. The short-term curves of storage modulus were recorded.

### 2.3. Creep Tests

The creep tests, based on the standard ISO 2285: 2019 [27], were carried out by the use of an Instron 9543 machine at a room temperature of 20 °C. Each test was kept for a short-term of 1800 s under various stresses, i.e., 0.2 MPa, 0.3 MPa, 0.4 MPa and 0.5 MPa. The strain versus time under various stresses were recorded.

### 2.4. General Theory

#### 2.4.1. Generalized Kelvin Model

**Figure 1:**
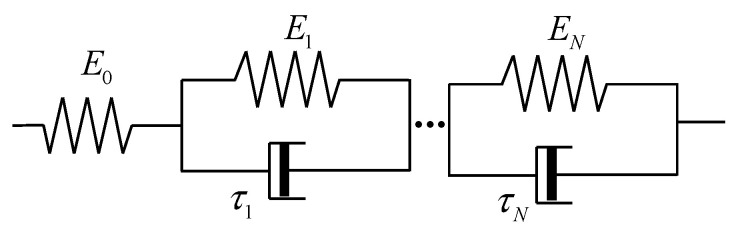
Composition of the generalized Kelvin model.

The Kelvin model is composed of a spring element and a dashpot element presented in parallel. The generalized Kelvin model is composed of an *N* Kelvin element and a spring element presented in series, as shown in Figure 1.

In Figure 1, it can be seen that the stress of the generalized Kelvin model equals the stress of every branch based on the characteristic of the series:

(1) $$\sigma =\sigma_{0}=\sigma_{1}=\sigma_{2}=\ldots =\sigma_{N}$$

When the stress is applied to the generalized Kelvin model, the corresponding deformation equals the total deformation of each branch. Thus, the strain of the generalized Kelvin model can be expressed as:

(2) $$\varepsilon =\varepsilon_{0}+\varepsilon_{1}+\ldots +\varepsilon_{N}$$

In addition, the *i*th Kelvin element is composed of a spring and a dashpot presented in parallel. Based on the characteristic of parallel, the stress of the *i*th Kelvin element contains two parts, which is presented as the following expression [14]:

(3) $$\sigma_{i}=E_{i}\varepsilon_{i}+\eta_{i}\frac{d\varepsilon_{i}}{dt}$$

where $\eta_{i}$ and *E_i_* are the viscosity and elastic modulus, respectively. *i* = 1, 2, …, *N*. Equations (1) to (3) represent the generalized Kelvin model.

When a constant stress $\sigma_{0}$ is applied to the rubber component, the creep occurs, and the strain can be expressed as [14]:

(4) $$\varepsilon =\frac{\sigma_{0}}{E_{0}}+\sigma_{0}\sum_{i=1}^{N}\frac{1}{E_{i}}\left(1-\exp(-\frac{t}{\tau_{i}})\right)$$

where *E*_0_ denotes the elastic modulus of the spring, $\tau_{i}$ denotes the relaxation time of the *i*th Kelvin element. According to Equation (4), the creep compliance is expressed as [21]:

(5) $$J\left(t\right)=\frac{1}{E_{0}}+\sum_{i=1}^{N}\frac{1}{E_{i}}\left(1-\exp(-\frac{t}{\tau_{i}})\right)$$

As for the generalized Kelvin model, the expressions of storage compliance *J*_1_ and loss compliance *J*_2_ are derived as follows [14,21]:

(6) $$J_{1}\left(\omega \right)=\frac{1}{E_{0}}+\sum_{i=1}^{N}\frac{1}{E_{i}+E_{i}\tau_{i}^{2}\omega^{2}}$$

(7) $$J_{2}\left(\omega \right)=\sum_{i=1}^{N}\frac{\tau_{i}\omega }{E_{i}+E_{i}\tau_{i}^{2}\omega^{2}}$$

where ω represents the angular frequency.

In the process of the DMA test, the storage modulus and loss modulus experimental curves are commonly obtained. In order to describe the experimental results, the expressions of storage modulus $E^{\prime }$ and loss modulus $E^{\prime \prime }$ should be derived. Based on the relationship between compliance and modulus [14], the dynamic moduli are expressed as:

(8) $$E^{\prime }\left(\omega \right)=\frac{J_{1}\left(\omega \right)}{J_{1}^{2}\left(\omega \right)+J_{2}^{2}\left(\omega \right)}$$

(9) $$E^{\prime \prime }\left(\omega \right)=\frac{J_{2}\left(\omega \right)}{J_{1}^{2}\left(\omega \right)+J_{2}^{2}\left(\omega \right)}$$

#### 2.4.2. Superposition Principle

This paper aims to obtain the converted creep behavior of CB-filled rubber based on the dynamic master curve. Therefore, the short-term DMA tests under various temperatures and frequencies should be carried out, and the master curve of storage modulus at a reference temperature *T*_ref_ should be constructed by use of the TTSP in the logarithmic time coordinate. In this process, the shifted distance is denoted as $\log\alpha_{T}$, which represents the temperature shift factor. According to the TTSP, the modulus *E* at various temperatures can be written as [28]:

(10) $$E(T,t)=E(T_{\mathrm{ref}},t/\alpha_{T})$$

where $\alpha_{T}$ is the temperature shift factor.

Similarly, as for the dynamic mechanical properties, the storage modulus and loss modulus can be expressed as [28]:

(11) $$E^{\prime }(T,\omega )=E^{\prime }(T_{\mathrm{ref}},\omega \alpha_{T})$$

(12) $$E^{\prime \prime }(T,\omega )=E^{\prime \prime }(T_{\mathrm{ref}},\omega \alpha_{T})$$

In order to verify the validation of the temperature shift factor, the WLF equation is often used to model the temperature shift factor. The WLF equation can be expressed as [29]:

(13) $$\log\alpha_{T}=\frac{-C_{1}\left(T-T_{\mathrm{ref}}\right)}{C_{2}+\left(T-T_{\mathrm{ref}}\right)}$$

where *C*_1_ and *C*_2_ are material parameters.

In this paper, the short-term creep tests are carried out under various stresses. As for stress-dependent cases, the WLF equation can be rewritten in another form, which is based on the time–stress superposition principle (TSSP). Similarly, the WLF equation of the TSSP can be written as [30].

(14) $$\log\alpha_{\sigma }=\frac{-C_{1}\left(\sigma -\sigma_{\mathrm{ref}}\right)}{C_{2}+\left(\sigma -\sigma_{\mathrm{ref}}\right)}$$

where $\alpha_{\sigma }$ is the stress shift factor, and $\sigma_{\mathrm{ref}}$ is the reference stress.

## 3. Results and Discussion

### 3.1. Dynamic Mechanical Property

**Figure 2:**
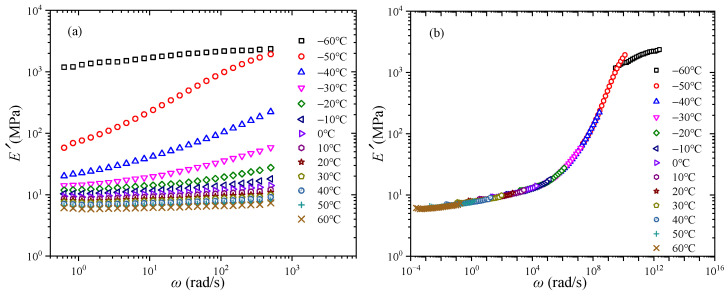
Storage modulus of CB-filled rubber: (**a**) experimental data, (**b**) master curve. Reprinted with permission from [25], Copyright 2025, Springer Nature.

In order to obtain the converted creep behavior of CB-filled rubber, the short-term DMA tests should be carried out. The storage modulus of CB-filled rubber under various temperatures is presented in Figure 2a. It can be seen that the storage modulus is frequency- and temperature-dependent. With the increase of temperature, the storage modulus decreases. The main reason is that when the temperature increases, the inner molecular chains have greater freedom of movement, and in the entanglement network, the entangled nodes’ number decreases [31]. Meanwhile, when the temperature is above 0 °C, the change of storage modulus is not obvious. In addition, in all cases, with the increase of frequency, it can be observed that the storage modulus increases. It has been reported that, as the frequency increases, the movement of the inner molecular segment of CB-filled rubber lags behind the change of external load and the inner consumption decreases, which results in the increase of storage modulus [32]. In order to investigate the long-term mechanical property, the master curve is commonly constructed on the basis of the TTSP. The master curve of storage modulus is presented in Figure 2b. The reference temperature is 20 °C. In Figure 2b, it is obvious that the short-term curves of storage modulus under various temperatures can be shifted to form a smooth master curve, which verifies the validation of the TTSP.

**Figure 3:**
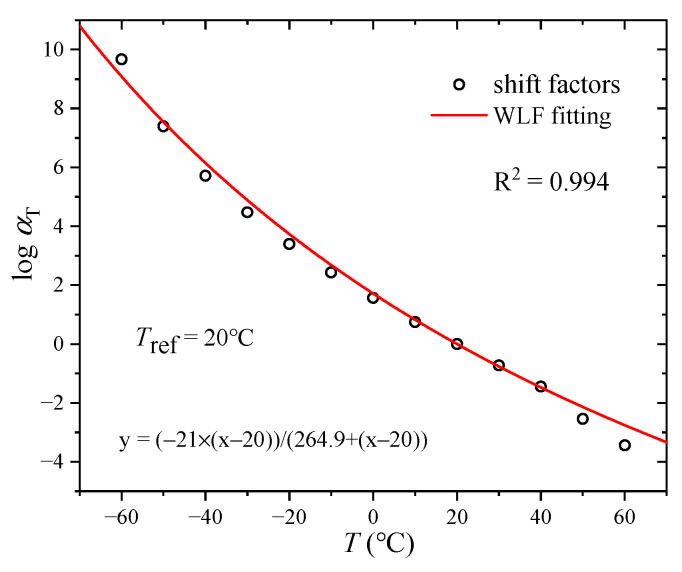
Time–temperature shift factor.

In the construction process of the master curve of storage modulus, the short-term curves of storage modulus under various temperatures are horizontally shifted to the storage modulus curve at the reference temperature in the logarithmic coordinate. The horizontal shift factors are obtained, as shown in Figure 3. It is well known that the shift factors are meaningful to the prediction of long-term mechanical property, which can be modelled by the WLF equation. In Figure 3, it can be seen that the WLF equation can describe the horizontal shift factors well, and R^2^ is 0.994. Meanwhile, the parameters of WLF are obtained. In other words, the curve of storage modulus at an arbitrary temperature can be derived by means of the WLF equation.

**Figure 4:**
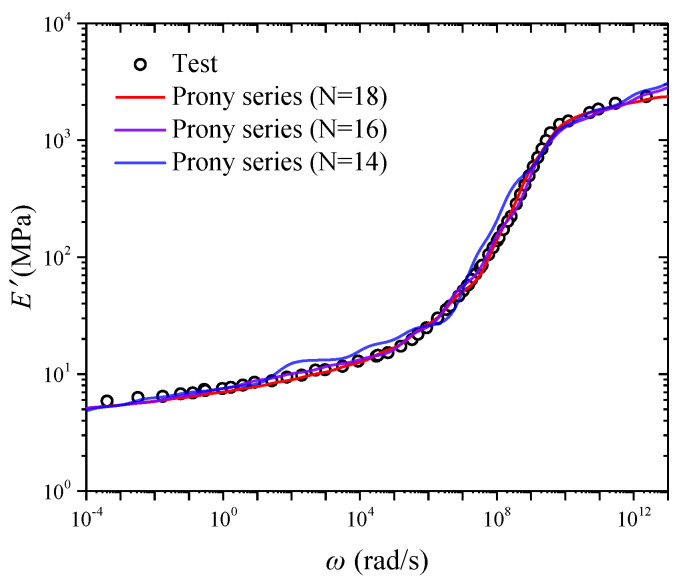
Simulation of long-term storage modulus by the generalized Kelvin mode. Reprinted with permission from [25], Copyright 2025, Springer Nature.

In order to obtain the converted creep compliance of CB-filled rubber, the master curve of storage modulus should be modelled by an appropriate viscoelastic constitutive model. In this paper, the generalized Kelvin mode is used to simulate the master curve of storage modulus. It is well known that the simulated ability of the generalized Kelvin model will be improved by increasing the number of the Kelvin elements [14]. However, an excessive number of Kelvin elements make the simulation process rather complicated. Therefore, the appropriate number of Kelvin elements should be determined. In addition, it has been reported that the direct mathematical fitting curve obtained by the generalized model often exhibits disadvantages [24], such as negative fitting parameters and fitting curve’s waviness. In this paper, the collocation method is used to avoid the above disadvantages. In Figure 4, it can be observed that the simulated ability of the generalized Kelvin model is improved by increasing the number of Kelvin elements. Meanwhile, when the number of Kelvin elements is 18, the fitting curve’s waviness can be avoided and R^2^ is 0.998, which verifies the superiority of the collocation method. Thus, the number of Kelvin elements in this paper is 18, and the parameters are presented in Table 1. It should be noted that increasing the number of target parameters may lead to a significant rise in the complexity of the fitting process. In addition, due to the mathematical principles underlying the collocation method, it is essential to predefine an appropriate relationship between frequency and relaxation time.

### 3.2. Creep Behavior

**Figure 5:**
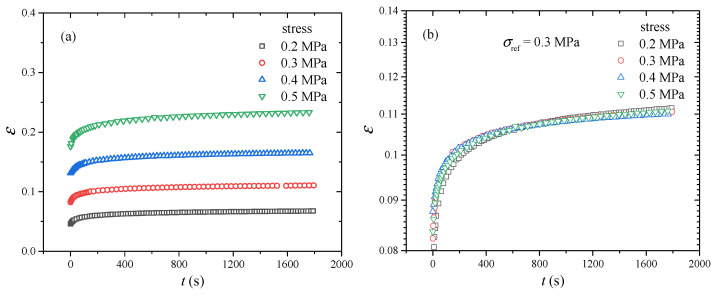
Creep under various stress: (**a**) experimental data, (**b**) master curve.

**Figure 6:**
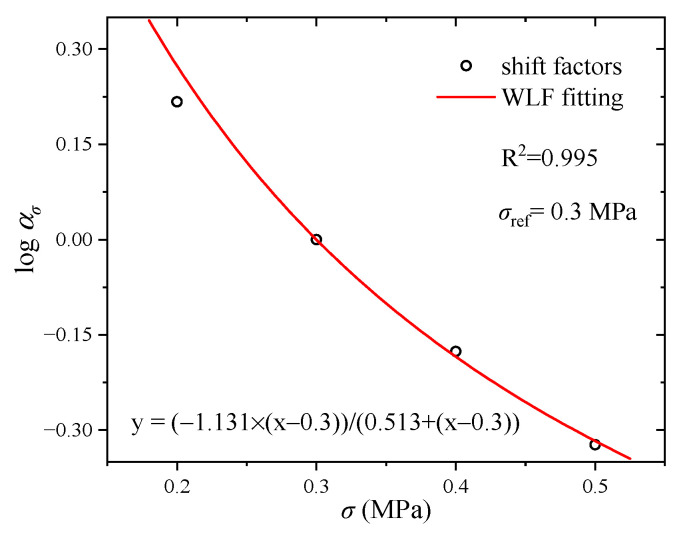
Experimental stress shift factor.

The creep results under various stresses (0.2 MPa, 0.3 MPa, 0.4 MPa, 0.5 MPa) are presented in Figure 5a. It can be seen that there are two obvious stages in all cases. In the first 200 s, the strain of CB-filled rubber increases rapidly. When the time is more than 200 s, the increase rate of strain is relatively low. In addition, it is obvious that the strain increases with the increase of stress. Comparing the creep curve under stress 0.2 MPa with the creep curve under stress 0.5 MPa, it can be observed that the higher stress needs more time to reach the equilibrium state. Meanwhile, it is noticeable that all strain curves are parallel to each other, which indicates that the creep curve at an arbitrary stress can be obtained by the use of vertical shift. Generally, the TTSP is commonly used to investigate the relationship between temperature and time. As for stress-dependent cases, the TSSP has been proposed [30]. In the construction process of the superimposed creep curve, the stress 0.3 MPa is regarded as the reference stress. The other creep curves under stresses 0.2 MPa, 0.4 MPa and 0.5 MPa are vertically shifted to the creep curve under stress 0.3 MPa, and a smooth superimposed creep curve is constructed in a logarithmic strain coordinate, as shown in Figure 5b. It can be observed that the creep curves under all stresses can be superimposed well, which indicates that the experimental results satisfy the TSSP. After the vertical shift process, the vertical shift factors can be obtained, as shown in Figure 6. It can be seen that there is an obvious nonlinear relationship between vertical shift factor and stress, which can be modelled by the WLF equation (Equation (14)). In Figure 6, it is obvious that the vertical shift factors can be modelled well by the WLF equation and the R^2^ is 0.995, which verifies the reliability of the TSSP.

**Figure 7:**
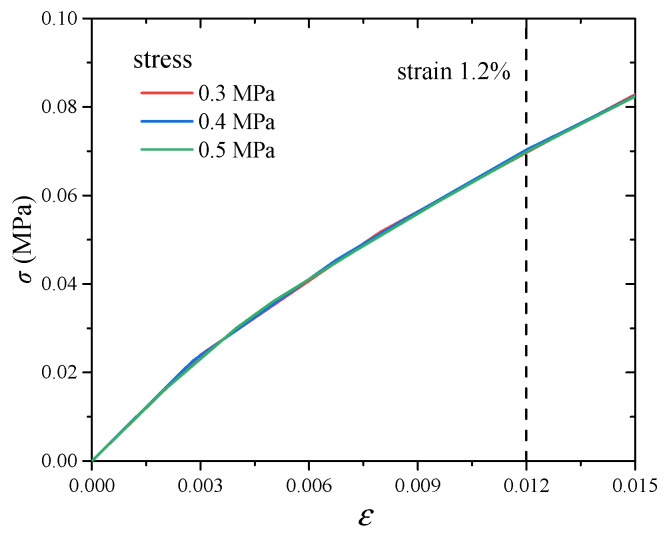
Experimental stress-strain curve.

**Figure 8:**
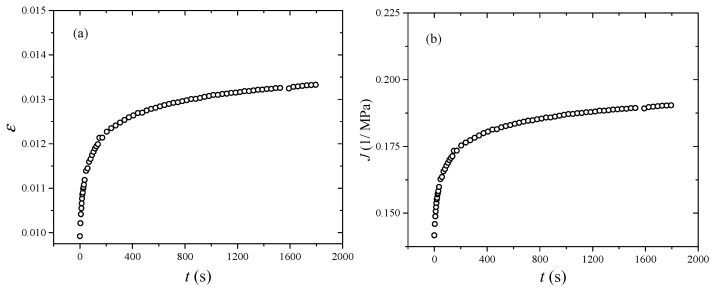
Creep behavior stress 0.07 MPa: (**a**) strain, (**b**) creep compliance.

As for the DMA tests, the dynamic strain amplitude is 0.2% and the prestrain is 1%. Thus, the maximum strain is 1.2%. According to the testing theory of the Gabo Eplexor 500N machine, the storage modulus is calculated by the maximum stress divided by peak strain. Therefore, the converted creep compliance curve corresponds to the stress when the strain is 1.2%. When a constant load is applied to the rubber component, the creep behavior occurs. Before the constant load keeping stage, there is a loading stage, and the stress-strain curve is recorded, as shown in Figure 7. When the strain is 1.2%, the stress-strain curves under various stresses (0.3 MPa, 0.4 MPa, 0.5 MPa) are coincident with each other. As the strain reaches 1.2%, the stresses are 0.0697 MPa, 0.0704 MPa and 0.0699 MPa, respectively. The average value of stress is 0.07 MPa. In addition, the creep test under stress 0.07 MPa is hard to obtain because of the precision of the loading machine. Therefore, the creep curve under stress 0.07 MPa is derived by use of the WLF equation (Equation (14)). After fitting the vertical shift factors by the use of the WLF equation, the equation parameters can be obtained, as shown in Figure 6. Substituting stress 0.07 MPa into the WLF equation, the vertical shift factor of stress 0.07 MPa can be obtained. After vertically shifting the creep curve of the reference stress 0.3 MPa by use of the obtained shift factor, the creep curve of the stress 0.07 MPa is obtained, as shown in Figure 8a. Based on Equations (4) and (5), the creep compliance curve of stress 0.07 MPa is calculated, as shown in Figure 8b. The calculated creep compliance curve will be used to verify the reliability of the converted result.

### 3.3. Creep Compliance Converted from Storage Modulus

In order to convert the creep compliance from storage modulus, the storage modulus should be modeled by Equation (8), and the parameters of the generalized Kelvin model should be identified. Because the direct mathematical fitting curve obtained by the generalized model often exhibits disadvantages [24], such as negative fitting parameters and fitting curve’s waviness, the direct mathematical fitting is not used to convert the creep compliance. In order to avoid the above disadvantages, it has been proved that the collocation method is very useful [20]. The role of the collocation method is to distribute the relaxation time, and a smoother fitting curve will be obtained when the relaxation time interval is smaller. Obviously, although the smaller relaxation time interval is useful, the fitting process is difficult because of the increasing Kelvin element. Based on the existing works, Park [33] proposed a scheme based on the equivalent time concept, and a logarithmic time interval less than 0.5 was recommended. In addition, it has been reported that the curve’s waviness is acceptable when the logarithmic time interval is less than 1 [34]. In this paper, the logarithmic angular frequency ranges from −4 to 13, as shown in Figure 4. If the logarithmic time interval is less than 1, the fitting process will become more and more difficult. Fortunately, it has been verified that the logarithmic time interval 1 is reliable [20]. Therefore, the logarithmic interval 1 is selected for the conversion of creep compliance. Because the logarithmic angular frequency ranges from −4 to 13, 18 Kelvin elements are used to describe the master curve of storage modulus in order to cover the whole frequency range of the experimental data. R^2^ is 0.998, which indicates that the fitting result is acceptable. Let $\tau_{i}=5\times 10^{i-14}s$, *i* = 1, 2, …, 18, which is based on reference [20]. The fitting process is finished by the use of the Levenberg–Marquardt optimization algorithm based on the 1 stOpt software. The fitting parameters of the generalized Kelvin model are obtained, as shown in Table 1. It can be seen that all parameters are positive, which avoids the disadvantage of the occurrence of negative fitting parameters.

**Figure 9:**
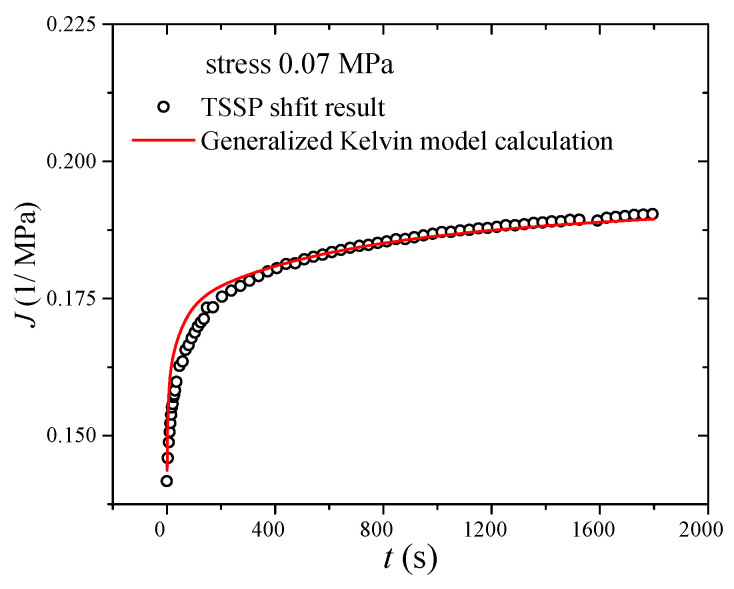
Creep compliance derived from the TSSP and converted from storage modulus.

After substituting the parameters into the creep compliance equation (Equation (5)), the creep compliance curve under stress 0.07 MPa is calculated, as shown in Figure 9 (red line). It is obvious that the converted creep compliance is in good agreement with the TSSP shifting result. In order to further verify the accuracy of the converted creep compliance, the RMSE value calculated over the entire experimental range is selected as an evaluation parameter. Generally, when the RMSE value is less than 0.5, the calculation result is acceptable. In this paper, the RMSE value is 0.0055, which indicates the reliability of the converted creep compliance.

## 4. Conclusions

It well-known that creep behavior is worthy of study for engineering because the rubber component often sustains loads for a long period. In order to obtain the converted creep behavior of CB-filled rubber, the short-term DMA tests has been carried out. It can be seen that the storage modulus is frequency- and temperature-dependent. In addition, the master curve is constructed on the basis of the TTSP in order to investigate the long-term mechanical property. Then, the creep compliance of CB-filled rubber has been converted from the master curve of storage modulus. On the basis of the collocation method, the generalized Kelvin model has been used to describe the master curve of storage modulus. It is found that the collocation method has the advantages of avoiding the fitting curve’s waviness and the occurrence of negative fitting parameters.

After substituting the fitting parameters of the generalized Kelvin model into the creep compliance equation, the creep compliance curve is calculated. The results show that the converted creep compliance is in good agreement with the TSSP shifting result, which indicates the reliability of the converted creep compliance.

## Figures and Tables

**Table 1:** Parameters of the generalized Kelvin model (Units: $\tau_{i}$/s, *E_i_*/MPa). Reprinted with permission from [25], Copyright 2025, Springer Nature.

*i*	$\tau_{i}$	1/*E_i_*	*i*	$\tau_{i}$	1/*E_i_*
1	5 × 10^−13^	5.833 × 10^−5^	11	5 × 10^−3^	1.613 × 10^−2^
2	5 × 10^−12^	7.937 × 10^−5^	12	5 × 10^−2^	1.587 × 10^−2^
3	5 × 10^−11^	9.016 × 10^−5^	13	5 × 10^−1^	1.493 × 10^−2^
4	5 × 10^−10^	4.684 × 10^−4^	14	5 × 10^0^	1.449 × 10^−2^
5	5 × 10^−9^	1.873 × 10^−3^	15	5 × 10^1^	1.389 × 10^−2^
6	5 × 10^−8^	1.204 × 10^−2^	16	5 × 10^2^	1.299 × 10^−2^
7	5 × 10^−7^	1.923 × 10^−2^	17	5 × 10^3^	1.250 × 10^−2^
8	5 × 10^−6^	2.500 × 10^−2^	18	5 × 10^4^	1.111 × 10^−2^
9	5 × 10^−5^	2.128 × 10^−2^	1/*E*_0_		4.212 × 10^−4^
10	5 × 10^−4^	1.694 × 10^−2^			

## Data Availability

The original contributions presented in this study are included in the article. Further inquiries can be directed to the corresponding author.

## Abbreviations

The following abbreviations are used in this manuscript:

CB	Carbon black
TTSP	Time-temperature superposition principle
WLF	Williams–Landel–Ferry
DMA	Dynamic mechanical analysis
